# Overcoming barriers for people with disabilities participating in income-generating activities: A proposed development framework

**DOI:** 10.4102/ajod.v12i0.1133

**Published:** 2023-03-20

**Authors:** Nokuthula Tinta, Unathi Kolanisi

**Affiliations:** 1 Department of Sustainable Food Systems and Development, Faculty of Natural and Agricultural Sciences, University of the Free State, Bloemfontein, South Africa; 2 Department of Consumer Sciences, Faculty of Science, Agriculture and Engineering, University of Zululand, Richards Bay, South Africa

**Keywords:** people with disabilities, barriers, income-generating activities, sheltered workshop, intervention strategies, effective participation, framework

## Abstract

**Background:**

People with disabilities in sheltered workshops are disempowered and face many barriers, adversely affecting their income-generating activities and weakening their competitiveness in the labour market. There is limited evidence on how to overcome these barriers.

**Objectives:**

This paper seeks to propose a framework to overcome the barriers experienced by people with disabilities participating in income-generating activities in a sheltered workshop.

**Method:**

The qualitative exploratory single case study was done with observations and semi-structured interviews as data collection methods. Purposive sampling was used to select 24 participants between ages 22 and 52 years, and content analysis was done of transcribed interviews. Community-based rehabilitation (CBR) guidelines were used to develop the framework.

**Results:**

A proposed framework was developed that outlined intervention strategies to address the barriers experienced by sheltered workshop participants to promote increased participation of people with disabilities in income-generation activities, thereby improving their quality of life.

**Conclusion:**

The participation of people with disabilities in income-generating activities is hindered by several barriers. However, the proposed framework overcomes the barriers to effective participation in income-generating activities.

**Contribution:**

People with disabilities will benefit from this framework as it will address their challenges and needs for empowerment. It would also inform stakeholders involved about these challenges and strategies.

## Introduction

Periodic global economic meltdowns remain a challenge that exacerbates many people’s vulnerabilities. The aftermath of numerous disasters such as floods, growing inflation and high unemployment that have recently hit South Africa, triggering economic downturns and volatility, has disrupted the country’s economic viability. The repercussions of this predicament have increased the likelihood of poverty (Jain et al. 2020). Regrettably, the detrimental implications of poverty are substantially worse among more vulnerable demographic groups, such as people with disabilities, than in non-disabled people. Similar to the general population, people with disabilities want to work productively to achieve their goals (Ofuani 2011). However, research shows that generally, people with disabilities are more likely to be resource poor, have lower employment rates, higher health care and living costs and less disposable income than non-disabled people (Banks, Kuper & Polack 2017; Gewurtz, Langan & Shand 2016; Mitra et al. 2017).

When the country’s unemployment rate rises, it creates greater competition for job positions and decreases the already limited space for people with disabilities to participate in the economy through employment. Unemployment negatively impacts their lives, and they ultimately have to depend on disability grants, family members and charity groups to sustain their livelihoods (Graham, Moodley & Selipsky 2013). People with disabilities are still not respected to the same extent as others to participate in economic development (Faizal, Kusnandar & Sulaeman 2020), contributing to this population’s disproportionately high unemployment rate. According to International Labour Organisation Statistics (ILOSTAT) (2022), their median unemployment rate stands at 7.6%, compared with 6.0% for people without disabilities. Even when employed, people with disabilities are more likely to be underemployed, earn less money and have fewer development opportunities than non-disabled people (Bonaccio et al. 2020). The United States Bureau of Labour Statistics (2021) states that people with disabilities are less likely to have completed a bachelor’s degree and higher than those with no disability. Among both groups, those who had attained higher levels of education were more likely to be employed than those who had attained less education. Income-generating measures become viable to explore as an alternative to paid employment for people with disabilities who are widely excluded from formal employment.

Empowering people with disabilities to generate income enables them to be financially independent and to integrate into society fully. Mpofu et al. (2011) remark that people with disabilities and their families need to be empowered to take care of their needs and take control of their lives and decisions, which leads to an improved future. Brisden (1998) posits that the economic empowerment of people with disabilities results in enhanced personhood and autonomy with economic benefits for society. Likewise, Eyben, Kabeer and Cornwall (2008) and Gathiram (2008) concur with the ideology of economic empowerment and argue that it optimises sustainability and people-centred development and creates less dependency on state resources. Economic empowerment maximises access to economic resources and opportunities such as employment, acquisitions of productive assets and skills development.

Employment discrimination is a reality of the labour market, and people with disabilities are more likely to be self-employed or unemployed than the general population (Mprah 2020; Pagán 2009). The literature demonstrates that poverty-eradication development strategies positively impact economic growth for the general population. Globally, income-generating activities in developed and developing countries help citizens become self-sufficient regardless of disability status (Singh & Chudasama 2020). However, studies show that people with disabilities are often excluded from poverty-reduction efforts, limiting their participation in economic activities, decision-making and social life (Ayoo 2022; Rodrik 2007; Singh & Chudasama 2020). According to Kallio (2019), while planning poverty reduction programmes and livelihood activities, context-specific local factors, target beneficiaries and market dynamics should be considered. Programmes should also incorporate aspects beyond mere income generation. Momsen et al. (2019) posit that intervention programmes should consider personal factors such as diagnosis, interpersonal skills (e.g. reading and writing) and job-related skills (Coelho et al. 2013; Harun et al. 2020).

The South African government has developed several policies and frameworks to address the barriers that prevent people with disabilities from fully participating in society. An article published by Tinta et al. (2020) which sought to highlight the barriers that limit the participation of people with disabilities in income generation activities (IGAs) in a sheltered workshop revealed that people with disabilities who work in sheltered workshops still encounter barriers that limit them from fully participating in IGAs. The barriers included the inability to use standard tools necessary for vocation, a lack of artistry skills, activities that are not stimulating, language and communication difficulties, a lack of funds and motivation. The understanding of barriers that limit economic participation for people with disabilities resulted in a need for effective intervention strategies to promote their participation (Tinta, Steyn & Vermaas 2020).

Numerous research studies have been conducted on effective economic empowerment interventions targeting people with disabilities. Tripney et al. (2015) examined such interventions to improve the labour market situation of adults with physical and sensory disabilities in low- and middle-income countries. The scholars could not conclude who, where or for whom interventions are likely to be effective and recommended a pressing need to engage in high-quality impact evaluations of interventions to encourage the involvement of individuals with disabilities in the labour market.

Groth and Söderström (2014), who explored how self-help facilitation provided by the organisation Response Network, can contribute to the empowerment of people living with disabilities in rural Zambia found that self-determination, education and participation contributed to the empowerment of people with disabilities. However, there is limited evidence regarding how the barriers that limit people with disabilities from effectively participating in income-generating activities in a sheltered workshop can be overcome. This paper aims to propose a development framework to overcome barriers that limit the full participation of people with disabilities partaking income-generating activities in a sheltered workshop.

## Conceptual framework

The theoretical framework of the study incorporated the motivational theory, the most widely accepted content theory of motivation (Maslow & Frager 1987) and the empowerment theory (Zimmerman 1995, 2000). The two theories were integrated into the study to review and outline the factors that affect the potential for entrepreneurship through income-generation activities. The Hierarchy of Needs theory highlights the levels for satisfying human needs (Maslow & Frager 1987). Empowerment theory is the complementary theory that aids in enhancing the actualisation of humans towards improved quality of life and well-being.

### Maslow’s hierarchy of needs

Maslow’s theory is founded on the premise that humans have hierarchically ranked needs. The most basic of Maslow’s hierarchy of needs are physiological needs that serve as the foundation for motivation. These are the needs for food, water, shelter and clothing that are necessary for the human body to function correctly. After the first level of needs is satisfied, safety and security become the driving force behind an individual’s behaviour. Safety needs are related to security, stability, dependency, protection, education and vocational training (McLeod 2020). Maslow believed that once a need has been met, it ceases to be a motivator. Then, the next level of need must be activated to motivate the person.

According to the Organisation for Economic Co-operation and Development’s Poverty Reduction Guideline (OECD 2001), economic, human, political, socio-cultural and protective capabilities are the five essential attributes of poverty. These five aspects of poverty are intricately linked and affect one another. Deprivation in one aspect typically leads to deprivation in other dimensions. Economic capacities are linked to food security, material well-being and social standing, including the ability to generate income and consume and own assets. Education, proper health and nutrition, safe drinking water and shelter are all critical to human well-being and form the foundation of human capabilities. Because disability is linked to poverty and dependency (Baffoe 2013), individuals could break the dependency cycle by participating in income-generating activities.

On the next level is a social need, which refers to the need to bond with other human beings, love and be a group member. Cultural conditions for belonging to a society, such as social standing or dignity, are critical for socio-cultural competencies and are highly prized by all of society. Socio-cultural skills affect people’s ability to participate as respected members of society, and socio-cultural capacities are more than just their connections and interactions. They also include social class, beliefs and values. The next level of human needs is esteem, which is the desire to be respected by one’s peers, feel important and be appreciated.

Finally, insecurity and vulnerability are important aspects of poverty intertwined with other aspects (OECD 2001). The fulfilment of esteem needs leads to self-confidence, strength and the capability to be useful in an organisation. The inability to fulfil these needs causes feelings of inferiority, weakness and helplessness, leading to social withdrawal. People with disabilities are often described as incapable, and their competencies are questioned in many societies (Baffoe 2013; Opoku et al. 2018); hence, people with disabilities desire to be acknowledged as full participants in society. After gratifying these needs, the higher-level need for self-actualisation emerges. The self-actualisation need describes individuals’ desire to realise their potential and achieve all they can.

### Economic empowerment

Economic empowerment for people with disabilities includes promoting opportunities to work and achieve self-sufficiency. This type of empowerment is vital to ensuring participation in the growth of society, especially in a country where poverty and inequality are firmly entrenched and promote independence, self-worth and economic power (Ofuani 2011).

Economic empowerment can be attained by giving people the tools they need to identify their problems and seek solutions (UNESCO 1993) and by offering them the chance to enhance their vocational knowledge and skills to find meaningful employment consistently. This will enable individuals to work together to create long-term social and economic prosperity (United Nations Economic & Social Council 2014). Increased productivity among working people with disabilities yields economic benefits (i.e. increased production of products and services) (Powers 2008) although evidence shows that opportunities are limited for persons with disabilities to access decent employment (Badmus 2017; Kidd et al. 2018).

Enhancing productivity, social inclusion and shared affluence require education and skills development. Education is an effective tool for economically empowering people with disabilities while simultaneously enhancing skills development, job opportunities and increasing access to economic resources (Ofuani 2011). People with disabilities, like people without disabilities, need specific skill sets to participate in productive economic activities and compete in the job market. Job market trends indicate that people with disabilities need assistance with education and training to improve their employment prospects (Munemo & Tom 2013). Therefore, developing relevant skills is a key component of the countries’ efforts to reduce poverty and utilise human resources.

Evaluating the skills needed to expand and promote the opportunity for people with disabilities to participate in economic activities is crucial (Powers 2008). Those who have had the chance to learn new skills have proven their ability to earn a living and assist society (Powers 2008).

Thus, economic empowerment ensures that people with disabilities have appropriate skills, capabilities, resources and access to sustainable income and livelihoods (Luttrell et al. 2009) and reduces reliance on government resources (Gathiram 2008). Necessary skills include technical, vocational, professional and entrepreneurial skills. Outcomes of training in vocational skills could yield self-directed employment and is an alternative to finding work to earn money. People with disabilities may benefit significantly from self-directed employment to gain financial independence.

Empowerment theory supports agency for change. Additionally, it enables communities to recognise injustice and inequality and increase the power of individuals who are perceived as helpless.

### The nexus of empowerment theory and income-generating activities

Empowerment theory provides valuable frameworks for fostering human empowerment for people with disabilities participating in income-generating activities. In order to promote state-independent, self-sufficient households and communities that can take care of themselves, communities use income-generating activities to effectively utilise the resources that are already accessible locally (National Development Agency [NDA] 2013). According to UNESCO (1993), income generation leads to gaining or increasing revenue. This does not imply that income-generating activities should always be seen to generate money or materials. Instead, monetary value attaches a quantifiable value to the services or products produced. National Development Agency (2013) further states that income-generating activities provide additional benefits, including: (1) reducing poverty, (2) improving the well-being of the communities, as well as (3) empowering and self-reliance. Income-generating activities generally assist economically disadvantaged populations. Thus, income-generating activities seek to improve the quality of life for all citizens and support human resource development.

In addition to providing a valuable framework for advancing human development, empowerment theory identifies practical strategies for combating oppression and boosting people’s strength, resiliency and resourcefulness. An empowerment theory includes processes and outcomes (Perkins & Zimmerman 1995; Sadan 1997; Zimmerman 1995, 2000). The empowering process includes structures, actions and activities to empower an individual. These processes enable individuals to develop skills and obtain resources to take charge of their decisions and solve problems (Moran et al. 2017). Outcomes consist of a measurable level of empowerment that an individual and organisation achieves as a result of an empowerment-focused intervention and newly acquired skills (Moran et al. 2017; Zimmerman 2000). Both processes and outcomes operate at numerous ecological levels (i.e. individuals, organisations and communities) and may emerge differently in various circumstances and populations (Zimmerman 2000).

The nexus between the IGA and economic empowerment is complementary and strengthens the household budget and the local economy by sustainably utilising locally available resources to empower people participating in IGA.

## Research method and design

### Study design

This paper builds upon an explorative single case study conducted on the barriers experienced by people with disabilities participating in IGAs (Tinta et al. 2020). The qualitative approach explored the experiences of people with disabilities participating in income-generating activities in a sheltered workshop.

### Setting

The study was conducted in a sheltered workshop managed and supervised by the Association for People with Disabilities in Bloemfontein, Free State, South Africa. The workshop only makes handcrafted products and sells them to the general public. The sheltered workshop was part of the University of the Free State service module the author is responsible for.

### Study population, sampling strategy and inclusion criteria

The study population consisted of 54 people with various impairments who participated in IGAs. The participants were women and men between 18 and 60 years who represented various ethnic groups and languages, such as Xhosa, Zulu, Sotho, Afrikaans and Tswana and who had been working in the sheltered workshop for more than 6 months were eligible for inclusion and purposefully selected (see Table 1). Only participants who had been registered at the sheltered workshop for more than 6 months were included in the study as they had a thorough understanding of the tasks carried out; hence, only 24 participants met the criteria and were selected.

**TABLE 1:** Participants’ socio-economic status.

Pseudonym	Gender	Age	Marital status	Home language	Level of education	Type of impairment	Employment status	Sources of income
A	Female	29	Single	Sesotho	Grade 8	Epilepsy	Unemployed	DG and stipend
B	Male	25	Single	isiZulu	Grade 9	Epilepsy	Unemployed	DG and stipend
C	Male	36	Single	Setswana	Grade 7	Dyslexia	Unemployed	DG and stipend
D	Male	43	Single	Setswana	Grade 9	Right hemiplegia	Unemployed	DG and stipend
E	Female	46	Single	Afrikaans	Grade 7	Learning disability and right hemiplegia	Unemployed	DG and stipend
F	Male	28	Single	isiXhosa	Grade 7	Sight impairment and musculoskeletal deformities	Unemployed	DG and stipend
G	Female	26	Single	Afrikaans	Grade 9	Spina Bifida	Unemployed	DG and stipend
H	Male	24	Single	Sesotho	Grade 7	Cerebral palsy	Self-employed	DG and stipend
I	Female	44	Single	isiXhosa	No schooling	Athetoid movement	Unemployed	DG and stipend
J	Female	36	Single	isiXhosa	Grade 7	Epilepsy	Unemployed	DG and stipend
K	Female	24	Single	isiXhosa	Grade 7	Right hemiplegia	Unemployed	DG and stipend
L	Female	22	Single	Afrikaans	Grade 9	Physical disability	Unemployed	DG and stipend
M	Male	27	Single	Sesotho	Grade 11	Learning disability and left hemiplegia	Unemployed	DG and stipend
N	Male	22	Single	Sesotho	Grade 9	Right hemiplegia (stroke)	Unemployed	DG and stipend
O	Female	52	Single	isiXhosa	Grade 2	Epilepsy and sight impairment	Unemployed	DG and stipend
P	Female	38	Single	Sesotho	Grade 7	Down syndrome	Unemployed	DG and stipend
Q	Male	46	Widow	Sesotho	Grade 7	Left-hand hemiplegia (stroke)	Unemployed	DG and stipend

DG, disability grant.

### Data collection

Observations and semi-structured interviews were used to collect data. Observations were conducted using an observational protocol that recorded data about the participant’s activities while making handmade products four times a week, for an hour and a half per day for two months. Creswell (2007, 2013) asserts that lengthy interaction with participants prevents drawing incorrect judgements. All the observations made throughout the sessions were recorded in field notes for later reporting and reflection.

Following the observations, participants were scheduled for one-on-one interviews. The participants agreed that as the sheltered workshop was convenient for them, all interview sessions could take place there. The researcher found Sesotho challenging to comprehend despite understanding and speaking English, isiXhosa and isiZulu well. As a result, a multilingual assistant who speaks Sesotho and English was employed. The researcher and the assistant translated the English-written interview schedule into Sesotho and isiXhosa, respectively, and both languages were used to conduct the interviews. The reason for translating this interview schedule into these languages was that most of the participants in the sheltered workshop spoke Sesotho and isiXhosa.

The interviews comprised short questions to elicit answers that reflected participants’ experiences with IGAs. Probing questions were utilised to get more information and elaborate on specific issues. The probes included rewording the question and requesting additional information (Bernard & Ryan 2010; Gobo 2008). The hour-long interview sessions were digitally recorded.

Saturation was reached, and data ceased with the 18th participants as no additional insight was identified. The researcher, therefore, conducted interviews with 18 of the 24 participants. According to Green and Thorogood (2004), 12–15 participants are deemed adequate for qualitative studies.

Trustworthiness was established according to the recommended strategies from Lincoln and Guba (1985). Credibility, dependability and transferability were used as guiding principles.

Credibility was achieved through using triangulation methods along with audio recording interview sessions. Dependability was promoted by using audiotapes and summarised transcripts. Enough information about the protective workshop and the participants was provided to ensure transferability, allowing them to assess how the circumstances and outcomes could be applied to their own experiences.

### Intervention categories of the proposed framework

The four main intervention categories of the proposed framework included education, health, livelihood and empowerment. These categories were plotted against the Community Based Rehabilitation (CBR) indicators outlined by the World Health Organization ([WHO] 2015) and Saran et al. (2019). The WHO recognises CBR as a comprehensive and multisectoral approach to equalising opportunities and involving people with disabilities in communities. To achieve complete inclusion and empowerment of people with disabilities, CBR served as a framework for intervention categories and subcategories. The subcategories of the intervention cover lifelong learning and skills development, support system, self-employment and finances and motivation. Some subcategories were modified and integrated with others to be context-based and address the barriers experienced by people with disabilities participating in income-generating activities.

### Data analysis

The researcher and the assistant transcribed recorded data in the original languages, Sesotho and isiXhosa. Before analysis, the transcribed data were translated into English. Two bilingual people confirmed the translations’ accuracy. Inductive content analysis was used to analyse the transcribed text (Ritchie et al. 2013) because it is a widely used qualitative research approach and scholars consider it a versatile way to analyse text data (Cavanagh 1997). The researcher perused the material and noted initial ideas to get familiar with the text. After identifying and coding pertinent items, which were then grouped into subcategories, ideas and concepts were identified. Conflicting theories and paradoxes were explored by presenting the data back to the participants for further exploration, and similar opinions were merged (Onwuegbuzie et al. 2009; Ritchie et al. 2013). Finally, the researcher interpreted the findings. Because there were few participants, data had to be manually analysed (Gobo 2008). Verbatim quotations from interview transcripts were extracted to illustrate relevant themes where appropriate.

### Ethical considerations

The researcher obtained permission to conduct the study from the workshop manager. The researcher established rapport with the participants to gain their consent and guarantee their anonymity (Ritchie et al. 2013). The participants were requested to give verbal consent as they did not want to sign the written consent form because they believed that signing any document would reveal their illiteracy. Verbal consent was obtained from the participants. All participants were informed that they had the right to decline to participate and could withdraw from the study at any time without any adverse consequences. Participants were assured that their names would be kept anonymous, that pseudonyms would be used when transcribing, and that all other identifying information would be stored securely and deleted after the study. The research instrument contained information that was not emotionally harmful to the participants. Ethical clearance to conduct this study was obtained from the University of Free State Research Ethics Committee (Ref No.: UFS-HSD2016/1436). This included outlining the procedures adopted for data generation, storage and use. The facilitator and the workshop manager were present for the debriefing. This was done to answer any queries the participants might have and to ensure there were no misunderstandings about what was expected of them. In order to maintain privacy, interview sessions were held one-on-one with participants in the dining room while other participants were involved with their daily activities.

## Findings and discussion

Two themes developed through analysis, namely ‘socio-economic status of the participants’, and ‘experiences with income-generating activities’. Two sub-themes under theme two are discussed: (1) the types of income-generating activities that participants were involved in and (2) the barriers that limited their participation.

### Socio-economic status of the participants

The first theme included information on the participants’ socioeconomic status, comprising information on their education level, employment status and sources of income.

### Education level

Six participants had completed their schooling up to the senior phase (grades 8–9), while the other eight had attended special schools up to the intermediate phase (grades 4–7). Only one participant completed grade 11, and one went to school through second grade.

### Employment status

Seventeen participants indicated that they were unemployed at the time of the study, mainly because of their disability and low school grades. A participant with cerebral palsy, who is self-employed outside of the workshop, explained as follows:

‘I never worked because of my grades, but I am self-employed, and I have a licence to drive.’ (H, male, age 24)

Three participants with epilepsy indicated that they were once employed, but their employment was terminated because of their disability. Woman with epilepsy, stated:

‘I worked as a cleaner; my teacher found this job for me but told me later to stop because of my sickness, so I never worked after that.’ (J, woman, 36 years old)

Participants’ statements identified their disabilities and level of education as reasons why they left work involuntarily in the past. Leonard Cheshire Disability (LCD), a leading provider of care and support to people with a wide range of disabilities, revealed similar findings in an unpublished baseline study (Kett 2012). These findings are consistent with the views documented in the literature (Gaunt & Lengnick-Hall 2014; Gold et al. 2012) that people with disabilities have high unemployment rates because of a lack of skills. The findings are also supported by Naami, Hayashi and Liese (2012), who noted that the unemployment rate for working-age people with disabilities in developing countries ranges from 80% to 90%.

### Sources of income

Sources of income were considered important as these revealed the type of capital a person possessed. The participants disclosed that they occasionally received a stipend from the workshop in addition to disability allowances as a source of income.

Participants reported that they were all recipients of a disability grant of R1600.00 per month from the government. For the purpose of this study, the government grant was regarded as an income. Seven participants also received a child support grant as an additional income. This finding suggests that these participants may escape poverty because of social grants. Studies in South Africa have found that social grants are beneficial in alleviating poverty (Gathiram 2008; Loeb et al. 2008; Samson et al. 2005), and grants are effectively targeted and provide income to households (Samson et al. 2005).

All the participants reported that they received a monthly stipend ranging from R40.00 to R200.00 from income-generating activities based on their attendance and when their products were sold. It was indicated in the background information that the stipend was intended to be an interim payment given to participants to assist them in covering some of their basic costs while they were at the workshop rather than a fixed income to support family obligations. A study by Koopman (2003) found that people with disabilities only receive an income if and when their products from income-generating activities were sold.

Four participants, primarily wheelchair users and sole breadwinners, stated that the disability grant money was insufficient as they had more needs than others without disabilities, as stated here by a woman with spina bifida:

‘I need to pay rent, food, clothes, school fees for my nephews, nappies for myself and shoes.’ (G, woman, 26 years old)

A single mother, of three children, with a physical disability added:

‘It is not enough, especially for us women; we have more needs like pads, you know.’ (I, woman, 44 years old)

The excerpts above show that a disability grant is used in households for other purposes rather than the intended person and that people with disabilities have more needs than people with no disabilities. Trani and Loeb (2010) found that households in Sierra Leone spend 1.3 times more on health care than non-disabled households, and people with disabilities have additional needs and expenses such as medicine, hearing devices and wheelchairs. Therefore, people with disabilities need more income in order to achieve the same goals as people without disabilities. According to Reith (1994), people with disabilities have to pay more for necessities like clothing, personal care products and house modifications that are seen as extravagances to people without disabilities. The findings demonstrate that sources of income for people with disabilities were insufficient when they needed to support others and themselves.

### Experiences of people with disabilities with income-generating activities in the workshop

The second theme identified is the experiences of people with disabilities with income-generating activities in the workshop. The subthemes that emerged from this theme were the types of income-generation activities the participants were involved in and the barriers that limit participation in income-generation activities. The latter subtheme is reported elsewhere in a previous publication titled *Barriers experienced by people with disabilities in income-generating activities: A case of a sheltered workshop in Bloemfontein, South Africa* (Tinta et al. 2020) and only summarised here.

### Types of income-generating activities the participants were involved in

Participants engaged in various income-generating activities. Six participants were doing beadwork such as necklaces and bracelets, eight were knitting scarfs, gloves and hats, two were sewing aprons and bags and the other two were making tapestry mats. The participants reported that they did not receive formal skills training but learned these skills informally from their co-workers at the workshop. This finding is consistent with LCD’s unpublished baseline survey (Kett 2012), which found that people with disabilities often learn skills through friends, family and informal training. These income-generating activities are considered outdated, in low demand in the modern market and have limited scope for business expansion (Kett 2012). These activities are seen as simple, within the means and capabilities of the participants, and requiring the least amount of human and non-human resources, such as skills, material and time to complete; they are among the most popular informal, traditional activities that people with disabilities partake in (Akyurek & Bumin 2017). This suggests that anyone can accomplish it, which clarifies why most participants engage in such activities. However, one can contend that despite being simple, prevocational training is still necessary for individuals to develop and master the skills needed to meet the labour market demands. In a study done by Naami (2010) on the impact of unemployment on women with physical disabilities in Tamale, Ghana, training in old-fashioned designs was noted as a barrier to employment faced by women with disabilities.

### Barriers that limit participation in income-generating

Participants noted several barriers that limited their participation in income-generating activities. The barriers include the inability to use standard tools necessary for the vocation, a lack of artistry skills, activities that are not stimulating, language and communication difficulties, a lack of funds and motivation.

One of the participants reported he struggled to utilise the sewing machine because of the nature of his impairment, while another participant stated his sight impairment made it difficult for him to select the beads he preferred, which impacted his performance. The researcher observed that some of the projects were constructed in a way that there was no prospect for growth and no practical framework for a long-term project goal. The products, such as those from knitting, tapestry and beading, were constructed unevenly, and there were inconsistencies in the construction of the patterns. It was also noted that seven participants failed to measure the fabric before cutting, leading to the fabric being wasted.

Nine participants asserted that the projects they participated in were not stimulating. As a result, they ended up wandering around or not finishing their work. Participants also noted that they were experiencing a language barrier with the facilitator because she only spoke English and Afrikaans. The participants did not comprehend English and primarily spoke Sesotho and isiXhosa. Their inability to adequately interact with the facilitator affected their participation as they could not articulate their goals.

All the participants unanimously reported that their biggest challenge was the lack of funds to purchase necessary supplies. This was evident as some of the participants were using tiny-sized beads that the community members had provided. The participants indicated that the only beads available were the ones they were using and were given scrap material to work with, and this hindered their full participation and resulted in poorly constructed products. The participants felt they were doing the same activities without any acknowledgement and positive reinforcement after completion.

The barriers mentioned above, directly and indirectly, limit the participation of people with disabilities in the income-generation activities and need to be overcome in order for them to participate in the labour market fully.

### Framework to counteract the barriers that limit the participation of people with disabilities partaking in income-generating activities in a sheltered workshop

A development framework is proposed for interventions to overcome the barriers that limit the full participation of people with disabilities partaking in income-generating activities. The development framework (Table 2) reflects CBR intervention categories and subcategories and barriers that limit the participation of people with disabilities in income-generating from the second theme of this research. Interventions are then proposed to reduce the barriers limiting the full participation in IGAs. Four out of the five main intervention categories in the CBR approach, namely education, health, livelihood and empowerment, were selected as relevant to the study. Proposed interventions may take the form of a device, programme, strategy or other types of action. The ideas for these interventions were drawn from literature (Khasnabis et al. 2010; Saran et al. 2021; Tripney et al. 2015; WHO 2015) and the author’s experience and involvement at the workshop.

**TABLE 2:** Intervention strategies to counteract barriers that limit the participation of people with disabilities partaking in income-generating activities.

CBR pillar categories	Intervention sub-category	Barriers that limit the participation of IGAs	Proposed interventions to reduce barriers
Education	Lifelong learning and skills development	Inability to use standard tools for vocation	Provide quality training programmes that include proper tool usage, use of scissors and simple activities such as basic tracing patterns, shapes, lines and cut exercises on a page
		Lack of artistry skills	Modifications to working equipment like sewing machines to accommodate various impairments
		Activities that are not stimulating	Provide skills development and training strategies, projects and initiatives to address developing human resources (Tripney et al. 2015). These can include art skills such as drawing and painting from real life, knowledge of materials, drawing and understanding proportion, colours and tone, mastering pencil control and brush strokes and creative skills
			Recruitment and training of specialised instructors to teach new skills sets that match the labour market requirements
			Offer multi-dimensional programmes that comprise various stimulating activities
			Provide vocational training for job opportunities
			Introduce modern market-driven projects
Health	Support system (assistive devices)	Language and communication difficulties	Employ instructors who are multilingual to overcome the language communication barrier Provide devices such as day calendars with symbol images for people with cognitive impairments (Saran et al. 2021). Provide sign language, interpreting services, speech software, speech and language therapy for people with speech impairments and Braille printing for people with sight impairments
Livelihood	Self-employment and finances	Lack of funds	Encourage the utilisation of reuse, reduce and recycle to create sustainable products Teach the target group how to use the resources surrounding them to be self-sufficient. This reduces dependency and promotes their right to self-determination. Teach the value of savings to reduce vulnerability and facilitate financial independence Implement financial awareness campaigns and market day as part of fundraising Financial participation in the intervention itself, such as stipends to cover the costs of attending training workshops (Tripney et al. 2015)
Empowerment	Motivation	Lack of motivation	Prioritise nurturing of innate skills to enable interiority and motivation
			Encourage decision-making to promote independence
			Plan and organise motivation programmes
			Celebrate achievements within the self-help organisation to boost or satisfy esteem needs
			Provide development and growth opportunities to satisfy self-actualisation needs

CBR, Community Based Rehabilitation; IGA, income generation activity.

## Conclusion

People with disabilities face barriers to full participation in income-generating activities at protective workshops, such as the inability to use standard tools necessary for the vocation, lack of artistry skills, language and communication difficulties, activities that are not stimulating, lack of funds and lack of motivation. Through this study, a framework was developed that proposes intervention strategies to counteract the barriers that limit the full participation of people with disabilities in income-generating activities in a protective workshop. Proposed strategies are categorised according to relevant CBR pillars for interventions that promote economic empowerment for people with disabilities. People with disabilities, instructors and organisations can use the framework to employ education, health, livelihood and empowerment intervention strategies to counteract the barriers and meet the needs of people with disabilities, increasing participation and improving the quality of life. Future research is needed to test the effectiveness of the intervention strategies and should be validated by people with disabilities.
